# A Novel Mobile App and Population Management System to Manage Rheumatoid Arthritis Flares: Protocol for a Randomized Controlled Trial

**DOI:** 10.2196/resprot.8771

**Published:** 2018-04-11

**Authors:** Penny Wang, Dee Luo, Fengxin Lu, Josephine S Elias, Adam B Landman, Kaleb D Michaud, Yvonne C Lee

**Affiliations:** ^1^ Division of Rheumatology, Immunology and Allergy Department of Medicine Brigham and Women's Hospital Boston, MA United States; ^2^ Digital Health Innovation Group Brigham and Women's Hospital Boston, MA United States; ^3^ Division of Rheumatology Department of Internal Medicine University of Nebraska Medical Center Omaha, NE United States; ^4^ National Data Bank for Rheumatic Diseases Wichita, KS United States; ^5^ Division of Rheumatology Department of Medicine Northwestern University Feinberg School of Medicine Chicago, IL United States

**Keywords:** arthritis, rheumatoid, symptom flare up, telemedicine, mobile applications

## Abstract

**Background:**

Rheumatoid arthritis flares have a profound effect on patients, causing pain and disability. However, flares often occur between regularly scheduled health care provider visits and are, therefore, difficult to monitor and manage. We sought to develop a mobile phone app combined with a population management system to help track RA flares between visits.

**Objective:**

The objective of this study is to implement the mobile app plus the population management system to monitor rheumatoid arthritis disease activity between scheduled health care provider visits over a period of 6 months.

**Methods:**

This is a randomized controlled trial that lasts for 6 months for each participant. We aim to recruit 190 patients, randomized 50:50 to the intervention group versus the control group. The intervention group will be assigned the mobile app and be prompted to answer daily questionnaires sent to their mobile devices. Both groups will be assigned a population manager, who will communicate with the participants via telephone at 6 weeks and 18 weeks. The population manager will also communicate with the participants in the intervention group if their responses indicate a sustained increase in rheumatoid arthritis disease activity. To assess patient satisfaction, the primary outcomes will be scores on the Treatment Satisfaction Questionnaire for Medication as well as the Perceived Efficacy in Patient-Physician Interactions questionnaire at 6 months. To determine the effect of the mobile app on rheumatoid arthritis disease activity, the primary outcome will be the Clinical Disease Activity Index at 6 months.

**Results:**

The trial started in November 2016, and an estimated 2.5 years will be necessary to complete the study. Study results are expected to be published by the end of 2019.

**Conclusions:**

The completion of this study will provide important data regarding the following: (1) the assessment of validated outcome measures to assess rheumatoid arthritis disease activity with a mobile app between routinely scheduled health care provider visits, (2) patient engagement in monitoring their condition, and (3) communication between patients and health care providers through the population management system.

**Trial Registration:**

ClinicalTrials.gov NCT02822521, http://clinicaltrials.gov/ct2/show/NCT02822521 (Archived by WebCite at http://www.webcitation.org/6xed3kGPd)

## Introduction

Rheumatoid arthritis (RA) is a systemic autoimmune disease that causes stiffness, swelling, and pain, resulting from inflammation in the joints [1]. Individuals with RA may experience occasional increases in inflammation that are associated with worsening symptoms called flares [2]. In focus groups, RA patients characterized flares as unpredictable, intense episodes that render them feeling helpless [3].

It is important for health care providers (HCPs) to monitor flares because frequent, long-lasting increases in inflammation can result in a permanent damage to joints and negatively impact quality of life [4,5]. The assessment of flares, however, is complicated. Flares are often unreported or inaccurately reported as RA patients may no longer recall flares that occurred between HCP visits. A study reported that 65% of RA patients who experienced flares were no longer facing issues by the time of their routine clinic visit [2]. In addition, patients and their HCPs frequently differ in what they call a flare. A qualitative research study, involving semistructured interviews and a Delphi exercise, revealed that, of the 10 domains identified as important by patients in the assessment of flare, only 4 overlapped with the domains considered important by their HCPs [6]. Although several research groups are working on the development of validated RA flare criteria, additional work is needed to develop appropriate scoring criteria and thresholds for flare severity and change [7,8].

To help HCPs and their patients better manage flares, better methods of tracking RA symptoms are needed. One potential method is through the use of mobile app. Nearly 64% of adults in the United States owned mobile phones in 2015, and 58.23% of mobile phone owners downloaded at least 1 health app [9,10]. Many types of apps have been developed to explore ways of helping people with chronic illnesses, such as diabetes and heart disease, and to monitor, understand, and manage symptoms [11]. Fewer apps have focused on the management of chronic autoimmune conditions, such as RA.

A research group in Japan recently created an app that enables RA patients to measure disease activity through patient-reported tender joints, a modified health assessment questionnaire, and measurement of gait balance using an accelerometer. In a pilot study of 65 RA patients, Nishiguchi et al demonstrated that patient-reported assessment of disease activity, via the mobile app, correlated with a validated measure of disease activity, the Disease Activity Score in 28 Joints, which includes physician-assessed joint counts and C-reactive protein, a serum inflammatory disease marker [12]. In a follow-up study in 2016 conducted by the same group, participants were surveyed about their opinions using the app. Overall, participants were favorable, stating that they had no or little difficulty recording their self-assessments [13]. Limitations, however, included the small sample size (N=9) and the limited length of follow-up (3 months). In addition, no information was provided regarding the use of these data by HCPs in medical decision making.

The *overall objective* of this proposal is to implement a mobile app plus a population management system to monitor RA disease activity between scheduled HCP visits over 6 months. The essential components in this system are as follows: (1) the mobile app, (2) the Web-based dashboard, and (3) the population manager. The Web-based dashboard consolidates incoming patient-reported data using preprogrammed algorithms to identify increases in disease activity, and the population manager is a trained individual who monitors the Web-based dashboard and connects patients with their HCPs. Our *hypothesis* is that the combination of mobile app and the population management system will improve patient satisfaction and management of RA disease activity by identifying RA flares as they occur and providing this information to the HCP through a trained population manager. The *rationale* is that the mobile app will increase patient involvement in disease assessment, whereas the population management system will support the integration of patient-reported data into the workflow of a busy clinical practice, enabling improved disease activity management between scheduled clinic visits.

## Methods

### Infrastructure

The setting for this study is the outpatient rheumatology clinic of an academic medical center in Boston, MA. This site was chosen because it is the home institution of the researchers involved in this study. The institution supports the development of mobile interventions through the Digital Health Innovation Group, which offers advice and assistance to ensure that the important standards for mHealth research (eg, cybersecurity, software design, and software maintenance) are met. The city of Boston, MA, is an ideal location for this study because it is one of the top 50 mobile-friendly cities in the United States [14]. In 2016, the availability of carrier networks in Boston was 51% above the national average of 81%. Upload and download speeds were 6.26 mbps and 8.74 mbps, respectively, and there were 2.12 mobile phone stores per 10,000 residents, enabling in-person customer service for mobile devices.

### Study Visit Design

#### General Schematic

This is a randomized controlled trial (RCT) of a mobile app plus the population management system. All participants will be assigned a population manager to serve as a contact person with whom participants can communicate regarding their flares. Participants randomized to receive the app will answer daily questions about their RA disease activity, using a validated self-report measure, Rheumatoid Arthritis Disease Activity Index-5 (RADAI-5), and the Patient-Reported Outcomes Measurement Information System (PROMIS) depression, fatigue, pain interference, physical function, and sleep impairment short forms [15,16]. The RADAI-5 was chosen because it is a short, 5-item questionnaire based only on patient-reported measures, whereas other common disease activity measures, such as the Clinical Disease Activity Index (CDAI) and Simplified Disease Activity Index, require in-person assessment of joints by a health care professional [17,18]. Studies have shown that patient-reported measures have similar, possibly greater, sensitivity in detecting treatment effects in RA [19].

The app will record the answers and summarize them in a graphical form to enable the visualization of trends. Population managers will have access to this information in real-time via a secure Web-based dashboard.

#### Inclusion and Exclusion Criteria

To be included in this study, participants must: (1) be diagnosed with RA by a board-certified rheumatologist, (2) be taking a disease-modifying antirheumatic drug (DMARD), (3) own a mobile device with an Android or iOS operating system, (4) be at least 18 years old, and (5) be able to speak English. Participants who do not plan on receiving follow-up care at this academic medical center will be excluded.

#### Randomization (Disease Activity Levels)

Participants will be randomized 1:1 using a publicly available Web-based randomization tool. Randomization will occur within categories stratified by disease activity, assessed by the CDAI: remission (≤2.8), low (>2.8-10), moderate (>10-22), and high (>22) [17].

#### Study Visits

**Figure 1:**
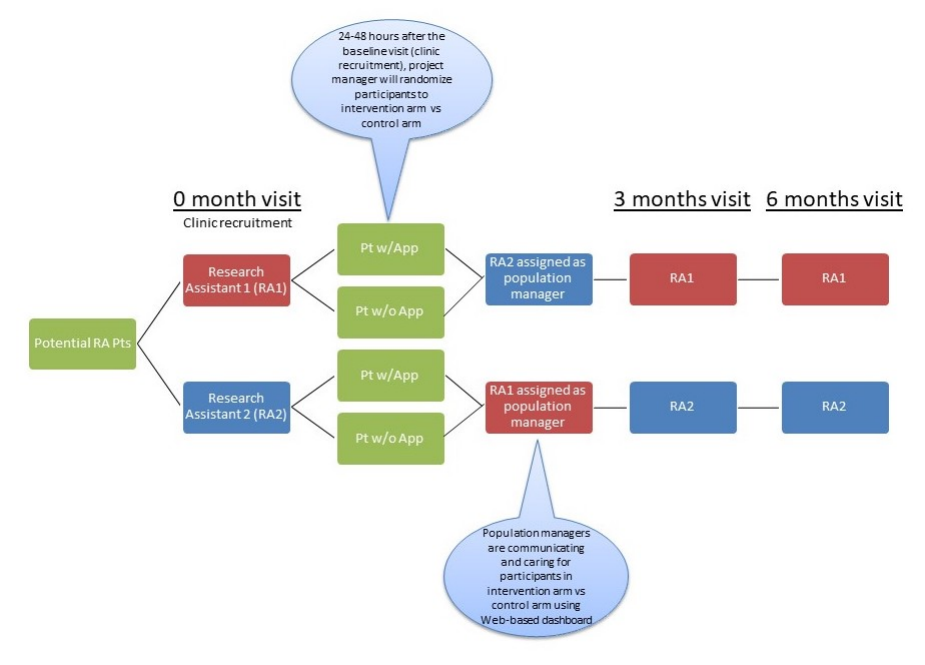
Study schematic showing the roles of research assistants at study visits versus the roles of population managers who communicate with subjects between study visits. Research assistants are blinded to the randomization, whereas population managers are not blinded. Pt = patient.

Study visits will occur at baseline, 3 months, and 6 months and will coincide with regularly scheduled visits to the participants’ rheumatology HCP. A trained research assistant will perform all assessments, including the joint examinations and tender point counts. Participants will also complete self-administered questionnaires to assess disease activity, flares, treatment satisfaction, and perceived efficacy of the patient-physician relationship. Specific data collection instruments are outlined in the section Data Collection. The research assistant will be blinded to the study arms to which the participants are assigned. This role is distinct from that of the population manager described in the section Population Management and Web-Based Dashboard (Figure 1).

#### Rheumatoid Arthritis Flare Study App

The mobile app was designed by the principal investigator and coinvestigators and custom developed by the ADK Group (Boston, MA), with guidance on the visual display from experienced app developers. Although RA patients were not involved in the design of this specific study or app, we incorporated information obtained from another study of a mobile phone app for RA patients, which was designed and led by one of the study co-investigators [20].

**Figure 2:**
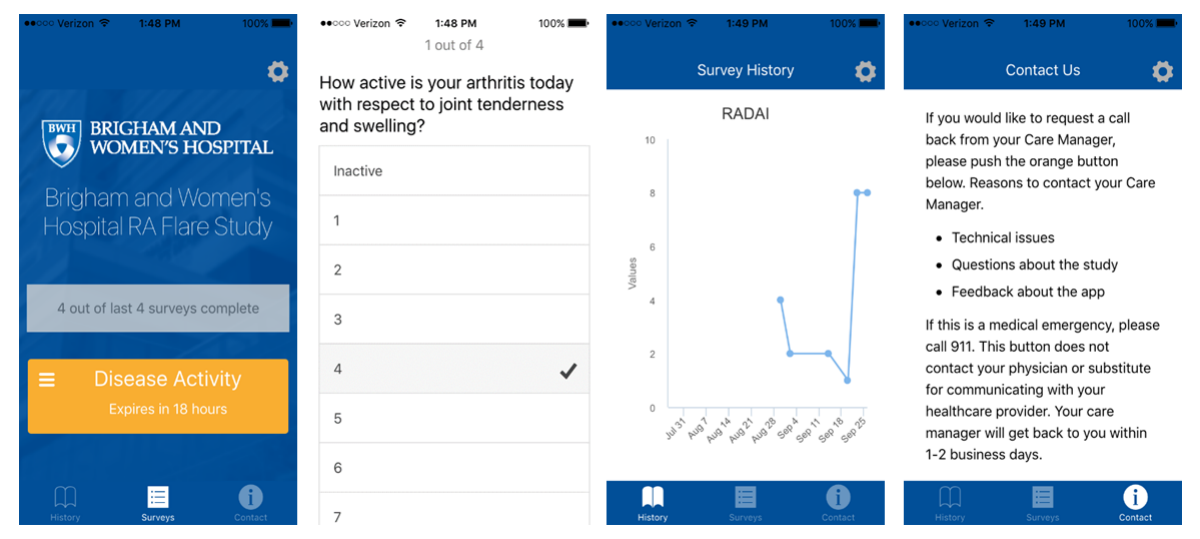
Screenshots of interface of RA Flare Mobile App.

All questionnaires used in the app have been validated and used in other studies including RA patients [15,16]. The app was designed to function on both the Android and iOS systems and met all guidelines for submission to the US Google Play and Apple App stores. Screenshots of the app are provided to show the visual interface (Figure 2).

At the first study visit, all study participants will be informed that, if they are randomized to receive the app, they will receive an email containing links to download the app from Google Play or the Apple App store. They will also receive a phone call from a trained study staff member, acting as a population manager, who will confirm that they were randomized to the intervention arm and take the participant through the process of downloading and using the app. Participants will register their account using a username, a password, and 5-digit pin number. After the app is downloaded, participants will be guided through a short interactive tutorial outlining important features of the app.

The app will send users a notification at 9:00 AM every day, prompting them to login using their username and 5-digit pin number to answer questions related to RA. If users have not answered their daily questions by 9:00 PM, they will receive another notification to remind them to complete the questions by 9:00 AM the next day. Once logged in, an option on the home screen will enable participants to select the daily survey. A table listing the surveys is provided in the section Data collection. Study participants will be able to view their responses in a graphic form on their apps if they are connected to the internet. However, no data will be stored on the app itself. Data will be transmitted securely and stored on secure servers at the study site. Data will be viewable by study staff on a password-protected Web-based dashboard, which includes graphs of all survey scores, as well as a table that shows the numerical scores. Additionally, the app will include a “Contact Us” button, which sends an email to the population manager, requesting further contact.

#### Population Management and Web-Based Dashboard

Each participant, regardless of study arm assignment, will be assigned to a population manager. The population manager is a trained study staff member who, unlike the research assistant performing visits, is not blinded to study group assignment. The population manager is responsible for communicating with participants during the course of the study. The initial call will occur immediately after the first study visit. The population manager will call to introduce him or herself and offer to assist participants with troubleshooting procedures to successfully download and register the app on the mobile device. The population manager will also call participants at weeks 6 and 18 to check in on participants and ask if they are experiencing an RA flare. These calls also serve to encourage participants to stay engaged in the study. If participants endorse a flare, an additional set of structured questions will be asked to assess the specific joints involved, as well as other symptoms and medication adherence.

**Figure 3:**
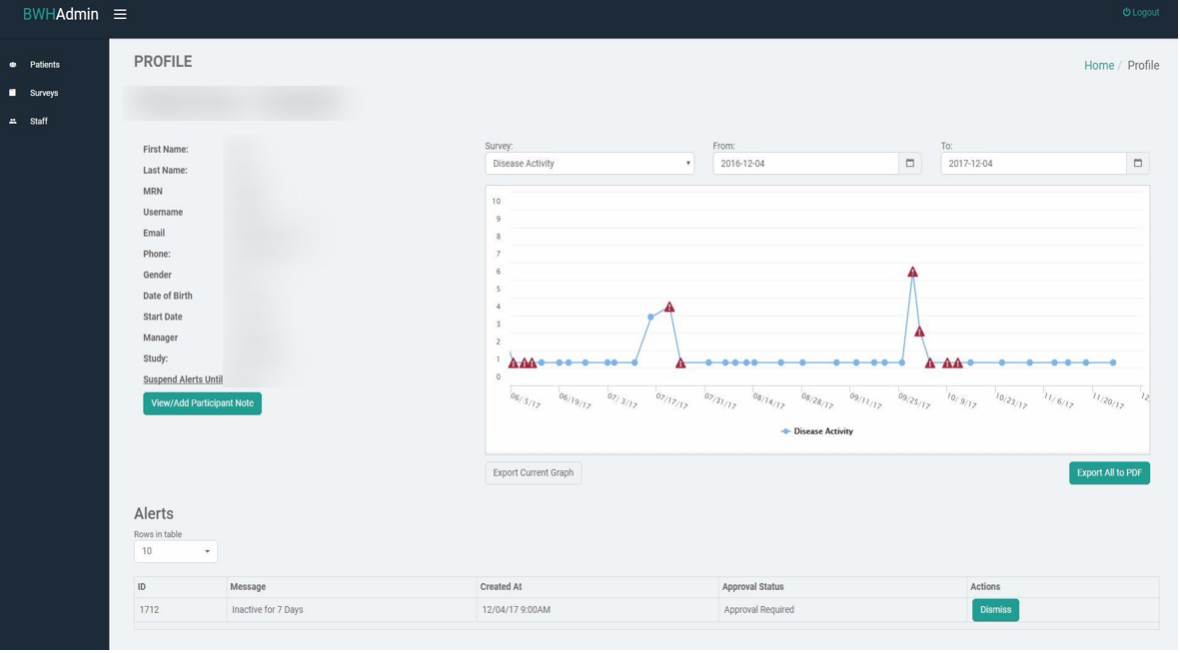
Web-based dashboard interface used by population managers to monitor participants’ responses to the app.

For participants randomized to receive the mobile app, the population manager will also monitor responses to the daily questionnaires on the Web-based dashboard (Figure 3). An algorithm will be used to identify potential RA flares by comparing the mean RADAI-5 score over the past 2 weeks to the mean RADAI-5 score over the previous 2 weeks.

**Figure 4:**
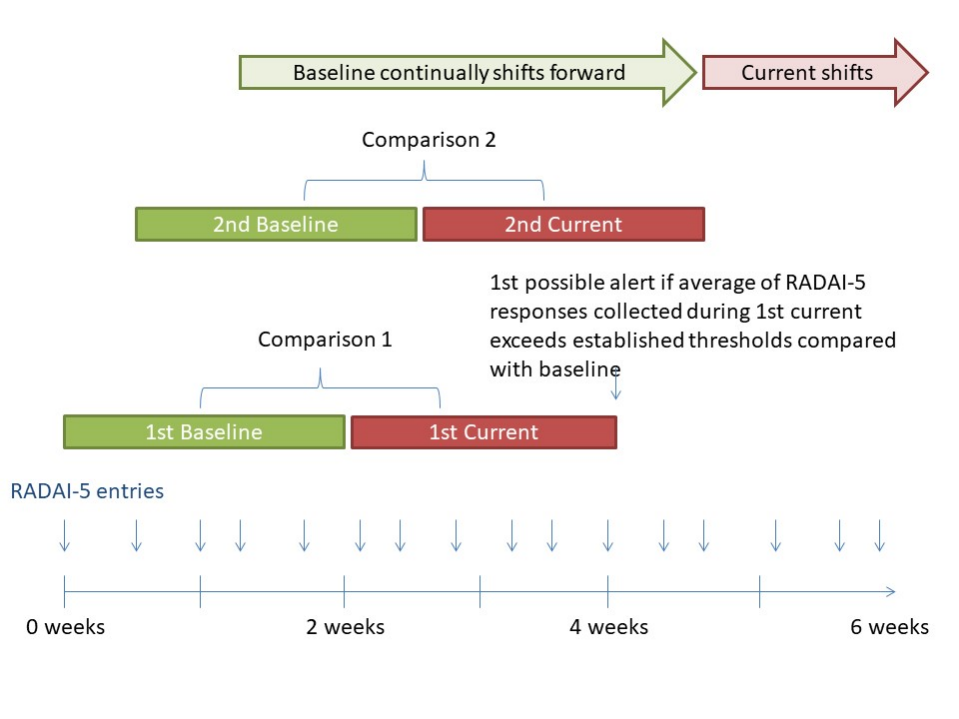
The flare algorithm compares Rheumatoid Arthritis Disease Activity Index-5 (RADAI-5) responses over a period of 2 weeks compared with the previous 2 weeks’ average. If the participant’s RADAI-5 score increases by more than 30% from the previous 2 weeks’ reading, the dashboard will generate an alert to the population manager.

If the participants’ RADAI-5 score increases by more than 30% from the previous 2 weeks’ reading and their current RADAI-5 score is >3, the dashboard will generate an alert to the population manager (Figure 4). The study team decided on this threshold after 1:1 conversations with board-certified rheumatologists at the study site. The appropriateness of this threshold will be evaluated as a part of this study.

The population manager will be responsible for communicating with study participants when the app algorithm identifies possible flares. The participant will be contacted and asked a structured set of follow-up questions about the possible flare, associated symptoms, and medication adherence. A 1-page summary will be compiled to send to the referring rheumatology HCP. Population managers will remind participants that their HCP is not required to respond to the summary, and it is up to their HCP to decide on appropriate follow-up procedures.

Although we considered prescribing a specific algorithm for HCPs to follow to address RA flares, we ultimately decided to allow each rheumatology HCP to respond to the notifications according to his or her own practice patterns. Our decision was based on 3 considerations: (1) there are no accepted guidelines regarding the treatment of RA flares, (2) surveys with the site’s HCPs indicated that physician participation would be negatively impacted by a rigid paradigm, and (3) we believe the mobile app plus the population management system will have broad applicability, independent of the specific management paradigm.

If participants do not answer any survey questions for 7 consecutive days, the population manager will contact the participant after the 7th day. Participants will be asked a set of questions to better understand the reasons underlying their lack of responses for the past week.

#### Data Collection

Data will be collected using the following methods: (1) all participants in the study will complete validated, self-reported questionnaires at study visits using paper forms (Table 1) and (2) participants in the intervention arm will also provide data daily through the app (Table 2). Rheumatology HCPs will also complete questionnaires at every study visit. Specifically, they will provide the physician global assessment of disease activity by answering the question, “Please rate your patient’s disease activity on a scale of 0-10 with 0 being very good and 10 being very bad” at every visit. At every visit following the baseline visit, HCPs will also be asked to complete the Physician Flare Questionnaire, which assesses their responses to flare notifications (eg, called patient, saw patient in person, changed medications; Multimedia Appendix 1). After the subject’s final study visit, his or her HCP will complete a Physician Exit Survey to provide feedback regarding his or her experiences with the mobile app and the population management system, including questions about its impact on patient-physician communication, overall management of RA disease activity, and physician workload (Multimedia Appendix 2).

**Table 1:** Evaluations occurring at all study visits among all study participants.

Type of assessment and instrument		Description
**Self-administered questionnaires**		
	Basic demographics (only during baseline visit)	Gender, age, ethnicity, race, education, and marital status
	Treatment Satisfaction Questionnaire for Medication (TSQM)	The TSQM asks participants about their level of satisfaction with their medications [21]
	Perceived Efficacy in Patient-Physician Interactions Questionnaire (PEPPI)	The PEPPI consists of 10 items asking participants about their self-efficacy in interacting with their health care providers [22]
	Patient global assessment of disease activity	Participants will be asked, “Considering all the ways in which illness and health conditions may affect you at this time, please rate how you are doing on a scale of 0-100 with 0 being very well and 100 being very poorly.”
	Brigham Rheumatoid Arthritis Sequential Study flare questions	Participants will be asked “During the past 3 months, have you had a flare in your rheumatoid arthritis (RA)?” If a participant answers yes, the participant will be queried about flare frequency, and how he or she treated the flare(s) [2]
	Flare Assessment of Rheumatoid Arthritis Questionnaire	This 13-item questionnaire was developed through a Delphi exercise to detect RA flares between medical visits [6]
	Patient Activity Scale II (PAS-II)	This is a validated index that assesses 3 American College of Rheumatology (ACR) Core Data Set patient-reported outcomes—physical function, pain, and global health [23]
**Assessments by trained study staff members**		
	Clinical Disease Activity Index (CDAI)	The CDAI is a measurement of RA disease activity calculated from the 28 joint count examination in addition to the physician global assessment and patient global assessment scores [17]
	Tender point examination	The tender point examination assesses tenderness at 18 sites and is used in the 1990 ACR Criteria for the Classification of Fibromyalgia [24]

**Table 2:** Self-administered questionnaires delivered through the mobile app between study visits among participants in the intervention group.

Instruments	Description	Frequency of administration
Mobile Entry Questionnaire	This module includes 4 items about how subjects feel about their connection with their health care team	Once at the beginning of the study
Modified Rheumatoid Arthritis Disease Activity Index (RADAI-5)	The RADAI-5 is a validated survey consisting of 5 items in a Likert format from 0 to 10 [15]: (1) How active was your arthritis in the last 6 months?; (2) How active is your arthritis today with respect to joint tenderness and swelling?; (3) How severe is your arthritis pain today?; (4) How would you describe your general health today?; and (5) Did you experience joint (hand) stiffness on awaking yesterday morning? If yes, how long was this stiffness?	The first question regarding arthritis activity in the last 6 months will be asked once a month. The last 4 questions regarding joint tenderness and swelling, pain severity, global health, and joint stiffness will be asked 3 times during an 8-day rotation
PROMIS^a^ Pain Interference Short Form	4 questions about the extent to which pain hinders engagement with activities [25]	Once every 8 days
PROMIS Physical Function Short Form	4 questions about the ability to perform basic activities [26]	Once every 8 days
PROMIS Fatigue Short Form	4 questions about tiredness and exhaustion [27]	Once every 8 days
PROMIS Depression Short Form	4 questions about negative mood and views of self [28]	Once every 8 days
PROMIS Sleep Disturbance Short Form	4 questions about sleep quality and restoration associated with sleep [29]	Once every 8 days
Flare treatment strategies	This module is based on questions asked in the Brigham Rheumatoid Arthritis Sequential Study preliminary study of flares in rheumatoid arthritis (RA). Specifically, this module will ask participants whether they had an RA flare in the 14 days and how they treated this flare [2]	Monthly

^a^PROMIS: Patient-Reported Outcomes Measurement Information System.

### Recruitment

The recruitment goal is 190 participants. Potential participants will be identified by searching for ICD-9 diagnosis codes for RA (714.x) in the Brigham Integrated Computing System scheduling database. Every week, study staff will generate lists of potential participants and prescreen patients via medical record review. Study staff will provide weekly lists to clinicians for their approval to contact the patients. Study staff will also be present in clinic during the busiest clinic sessions to remind HCPs of potential participants and to facilitate referrals. Every eligible participant will review the informed consent document approved by Partners Institutional Review Board, and written informed consent will be obtained by a trained research assistant. All study staff will complete training in HIPAA and Ethics in Human Subjects Research. Approximately 50.0% (95/190) will be randomized to receive the mobile app (intervention group). On the basis of attrition rates from previous studies, we expect ≥61.0% (122/190) of subjects to complete the study.

### Outcome Measures

To assess patient satisfaction, the primary outcomes will be scores on the TSQM and PEPPI questionnaire at 6 months [21,22]. To determine the effect of the mobile app on RA disease activity, the primary outcome will be CDAI at 6 months [17].

The TSQM is a validated questionnaire that assesses medication satisfaction [21]. The different components of medication satisfaction are the following: side effects, effectiveness, convenience, and overall satisfaction. There are 14 questions, scored on a 5- or 7-point Likert scale. Responses to each question are assigned a score between 0 and 100, with 0 being extremely dissatisfied to 100 being extremely satisfied. The TSQM was initially validated in a national study of chronic disease, including patients with arthritis, asthma, depression, hyperlipidemia, hypertension, migraine, and psoriasis. It was subsequently validated in separate populations of patients with multiple sclerosis, cystic fibrosis, and coronary disease [30-32]. In one study, ceiling and floor effects were observed in the side effects domain [32]. Of note, if these effects are a problem, a separate score (TSQM-9) can be calculated, removing the 5 items in the side effects domain. This abbreviated score was shown to have good construct and convergent validity, along with high internal consistency and good test-retest reliability [33]. In a study of individuals with chronic illnesses including 25.6% (44/172) RA patients, representative scores on the TSQM were 77.1 (SD 25.2) for side effects, 61.6 (SD 24.16) for effectiveness, 66.1 (SD 17.1) for convenience, and 68.8 (SD 20.6) for overall satisfaction [34]. Although no studies have specifically examined the responsiveness of the TSQM in RA, a study of osteoarthritis patients revealed that perceptions of convenience, effectiveness, and overall satisfaction increased with treatment with a nonsteroidal anti-inflammatory drug [35]. The TSQM has also been used in clinical trials of RA patients to compare patient satisfaction with the effects of different DMARDs [36].

The PEPPI is a validated questionnaire that assesses self-efficacy in the patient-physician interaction [22]. It consists of 10 questions, scored on 5-point Likert scales, reflecting very confident to not confident at all. In multiple studies of elderly individuals and patients with osteoarthritis, the PEPPI showed construct, convergent, discriminant, and structural validity [22,37,38]. Although a ceiling effect was noted in the PEPPI 10-item scale, no ceiling effects were noted using the abbreviated 5-item scale, and no floor effects were noted for either scale [37-39]. Of note, the PEPPI was recently used in a 12-month RCT of an eHealth interactive self-assessment website (Sanoia) in RA [40]. The 320 participants in this study were similar in age, sex, and disease duration to the population we are recruiting. The mean baseline PEPPI score was 39.2 (SD 7.8). Participants randomized to receive the active intervention had small increases in their PEPPI score (mean 0.6, SD 5.2), whereas participants randomized to the control arm had concurrent decreases in their PEPPI score (mean ‒-0.91, SD 6.08). Although the magnitude of change was small, the difference between the 2 groups was statistically significant (*P*=.01), indicating that this instrument is able to detect differences between an active self-monitoring intervention and control.

### Statistical Analysis

The primary analysis will be a completer analysis. Summary statistics (eg, frequencies, means, and medians) of the baseline variables will be calculated to assess the effectiveness of randomization. Treatment effects (mean differences in outcomes between the 2 groups at 3 and 6 months) will be estimated with mixed models. This model includes the interaction of treatment and time and allows adjustment for baseline scores. For each outcome measure, we will also adjust for age, sex, race, disease duration, and any variables that are statistically different from each other in unadjusted analyses (*P*<.10). A 2-tailed *P* value of .05 will be considered significant. All analyses will be conducted by a blinded statistician using SAS 9.3 (SAS Institute).

On the basis of previous studies performed by this research group within this population, we do not expect a large amount of missing data in the primary outcome measures and covariates. These data are obtained during in-person study visits. A research assistant is present during all study visits and checks forms for completion. When necessary, multiple imputation by chained equations will be used to impute missing variables.

### Sample Size Calculation

A total of 2920 RA patients were seen at the site of recruitment between April 30, 2013, and May 1, 2014. Of these patients, 1980 were prescribed at least one DMARD between April 30, 2013, and May 1, 2014, and approximately 60.00% (1188/1980) had moderate-to-high disease activity. From the 2013 Pew Internet Tracking Survey, we estimate that 50% of these patients (594/1980) own mobile phones [41]. Of these 594 individuals, we expect 80.1% (476/594) to meet the remainder of the inclusion criteria (≥18 years old, English speaking), and 40.0% (190/476) to agree to participate.

On the basis of previous studies examining differences in patient satisfaction, the expected difference between groups is 9.4 (SD 18.4) in TSQM scores [42,43]. No data exist regarding the expected difference in CDAI scores for this intervention. However, the minimal clinically important difference in CDAI scores is 12 [44,45]. On the basis of attrition rates from previous studies, we expect ≥61% (122/190, 64.2%) subjects to complete the study. Given a sample size of 122 subjects and an alpha level of .05, we will be able to reject the null hypothesis that the population means of the experimental and control groups are equal with probability (power) 0.80 to 0.99.

### Fidelity of the Intervention

In addition to the assessments of satisfaction and efficacy described above, we will also assess the fidelity of the intervention. Systems are in place to monitor survey completion rates, and an alert is issued to the population manager if participants have not answered a survey in the past 7 days. Information is also obtained on the exit survey to determine whether participants were able to download the app.

## Results

Recruitment is currently underway. We started patient recruitment in November 2016 and will continue until the recruitment goal of 190 participants has been achieved. Findings on the study’s primary outcomes are expected to be finalized by September 2019. Thus far, user feedback from both RA patients and HCPs has been positive. RA patients have particularly enjoyed the ability to track their disease activity and symptoms and to share these results, using the app’s graphic interface, with their family members. HCPs have appreciated receiving information about their patients between regularly scheduled clinic visits.

## Discussion

### Summary and Strengths

The findings from this study will represent the first randomized trial to test the effects of a combined mobile app and the population management system on a patient population with RA. Although several mobile apps exist to monitor symptoms of chronic disease, none have incorporated the use of a population management system for the management of disease activity in RA. This manuscript was written meeting the checklist to report health interventions using mobile phones [46].

In a systematic review of mobile apps to assist RA patients in disease management, Grainger et al reported that high-quality apps, including monitoring tools that assess disease activity using validated instruments, are lacking [47]. They also noted the importance of delivering the tools via a user-friendly interface. Our medical team collaborated with the local app developer, as well as other experienced developers, to address these 2 issues. First, we worked with the local app developer to incorporate a validated measure of self-reported RA, the RADAI-5, and PROMIS assessments of pain interference, physical function, fatigue, depression, and sleep disturbance. Second, we met experienced app developers to get advice regarding the user interface during the development stages. The developers provided valuable feedback, including suggestions to create large menu buttons at the bottom of the screen rather than to rely on a list of menu options as a dropdown box along the side. As a result, we were able to develop a mobile app that incorporates useful information for physicians, while also being easy for patients to enter and visualize data.

We foresee tangible benefits to both patients and HCPs. For patients, an app can be beneficial by providing tools to easily track and record their disease trajectories. If successful, this system may spark greater patient engagement in self-management of RA. People with chronic diseases have been more engaged in using mobile apps in managing their diseases than people managing other aspects of their health [48]. For HCPs, an app and a population management system may be beneficial by providing a more complete picture of their patients, beyond the snapshots they currently see during scheduled clinic visits. We specifically created the combined app and population management system so that it does not create a large amount of work for busy HCPs. Instead, we maximize the use of other trained staff members (eg, population managers) and only involve HCPs when clinically significant events are identified by flare alerts. This increased reservoir of information may then serve as a catalyst for more effective patient-HCP communication. A formal cost assessment was not performed as this is a research study designed to assess feasibility and efficacy, and the costs involved in this endeavor will likely differ from actual implementation in the clinic setting.

### Barriers and Limitations

Many barriers have occurred during app development and study implementation. Specifically, beta-testing took more time than we planned, and, as a result of beta-testing, additional revisions were required. After the app was developed, regular maintenance and updates were required. These included testing and updates for new releases of the iOS and Android operating systems and cross-device and browser testing for new device and browser versions. In addition, implementation of the study was disrupted for a period of 2-6 days when surveys were not sent to participants due to disruptions in server access. These difficulties highlight the importance of ensuring that timelines include appropriate consideration for beta-testing, and budgets include appropriate resources for support and maintenance activities.

A major limitation is that this system is not integrated with the existing electronic medical record (EMR). Although we originally sought to integrate this system, multiple factors intervened. Specifically, the hospital system changed EMRs during the planning phase of this study. Thus, the institution’s information technology priorities were focused on the clinical implementation of the new EMR, not on research endeavors. In addition, as a result of the transition in EMRs, HCPs were required to complete multiple training sessions and change their workflow to accommodate the new EMR. As a result, additional meetings with HCPs to discuss the best strategies for integration into the EMR were not possible at this time. The ability to translate this pilot study to a large-scale clinical implementation will depend on integration with the EMR. Although technically possible, integration with the EMR will require much negotiation with hospital administration and HCP groups, as well as an appropriate budget to fund the processes to support this integration.

In addition, we expect a fair amount of missing data from the app due to varying engagement from participants. However, our primary objective is not to use these data in statistical analyses; rather, our intention is that these data be useful to patients in monitoring and understanding their disease. Even if patients do not enter 50% of the data, they will still, on average, have at least 1 data point for disease activity per week and at least 1 data point for each of the other symptoms every 2 weeks. These data, in themselves, can be helpful and are more than is normally obtained without the aid of a disease activity and/or symptom tracker. We will also analyze the completion rates of daily surveys as proxies for patient engagement and overall feasibility.

Other limitations include the following: (1) the possibility of a ceiling effect if most participants have low disease activity and score well on the primary outcome measures, even at baseline; (2) the study being available only to English speakers, as not all of the surveys have been validated in other languages; and (3) limited generalizability, as we are only enrolling individuals who own a mobile device and, therefore, are likely receptive to mobile technology. In a study of 9183 participants in the Arthritis Internet Registry, older age and lower income were significantly associated with lower rates of mobile phone ownership [20]. However, recent studies suggest that mobile phone ownership is growing at a rapid rate, even among individuals over 50 years. On the basis of a 2016 report by the Pew Research Center, 74% of individuals between 50 and 64 years of age now own a mobile phone, a number which is up from approximately 50% in 2013 [49,50]. Similarly, mobile phone ownership in low-income households (<US $30,000/year) now exceeds 50% and continues to rise [51].

### Conclusions

The successful implementation of a mobile device and population management system may help integrate patient-reported data into the workflow of a busy clinical practice. We expect this model to be easily adaptable to other rheumatic conditions, as well as nonrheumatic chronic illnesses. The PROMIS questionnaires used in this study were specifically developed to be relevant across different conditions for the assessment of symptoms and functions, and we anticipate that a mobile app combined with a population management system will have broad applicability in helping patients better understand their disease, improving patient-HCP communication and, ultimately, improving disease management and overall satisfaction in their care.

## Appendix

Multimedia Appendix 1 Physician exit form.

Multimedia Appendix 2 Physician study visit follow-up questions.

## Abbreviations

CDAI: Clinical Disease Activity Index
DMARD: disease-modifying antirheumatic drug
EMR: electronic medical record
HCP: health care provider
PEPPI: Perceived Efficacy in Patient-Physician Interactions
PROMIS: Patient-Reported Outcomes Measurement Information System
RA: rheumatoid arthritis
RADAI-5: Rheumatoid Arthritis Disease Activity Index-5
RCT: randomized controlled trial
TSQM: Treatment Satisfaction Questionnaire for Medication
