# Supplementary material for: A Novel Mobile App and Population Management System to Manage Rheumatoid Arthritis Flares: Protocol for a Randomized Controlled Trial
Source: JMIR Res Protoc. 2018 Apr 11;7(4):e84. doi: 10.2196/resprot.8771 (PMC5917083; doi:10.2196/resprot.8771)
Supplement: Multimedia Appendix 1 [file resprot_v7i4e84_app1.pdf]

PHYSICIAN STUDY VISIT Follow Up QUESTIONS

1. Please rate your patient's disease activity on a scale of 0 - 10 with 0 being very good and 10 being very bad: ____

DELETE THIS PORTION IF PATIENT DOES NOT HAVE APP

During the last 3 months, you received **NUMBER** flare report(s), which is attached to this email for your reference. Please answer the following regarding the flare report(s):

2. The flare report assisted me in the management of my patient:
    - a) Strongly Agree
    - b) Agree
    - c) Neutral
    - d) Disagree
    - e) Strongly Disagree

3. Please check any action that was taken in response to the flare reports received in the last 3 months:
    - a) No action required
    - b) Called patient
    - c) Saw patient in person
    - d) Changed medication
