# Supplementary material for: A Novel Mobile App and Population Management System to Manage Rheumatoid Arthritis Flares: Protocol for a Randomized Controlled Trial
Source: JMIR Res Protoc. 2018 Apr 11;7(4):e84. doi: 10.2196/resprot.8771 (PMC5917083; doi:10.2196/resprot.8771)
Supplement: Multimedia Appendix 2 [file resprot_v7i4e84_app2.pdf]

ID:
Date:

Physician Exit Form

Please indicate your level of agreement with the following statements.

1. The smartphone app + population management system improved patient-physician communication.

□ Strongly disagree
□ Disagree
□ Neither agree nor disagree
□ Agree
□ Strongly agree

2. The smartphone app + population management system resulted in earlier changes to long-term DMARD therapy.

□ Strongly disagree
□ Disagree
□ Neither agree nor disagree
□ Agree
□ Strongly agree

3. The smartphone app + population management system improved overall management of RA disease activity.

□ Strongly disagree
□ Disagree
□ Neither agree nor disagree
□ Agree
□ Strongly agree

4. The smartphone app + population management system increased my workload.

□ Strongly disagree
□ Disagree
□ Neither agree nor disagree
□ Agree
□ Strongly agree

5. I would like to continue offering this system to my patients.

□ Strongly disagree
□ Disagree
□ Neither agree nor disagree
□ Agree
□ Strongly agree

6. If you have any additional comments or suggestions, please provide them here:
